# A case of palatine tonsillar metastasis of lung adenocarcinoma: A CARE-compliant article

**DOI:** 10.1097/MD.0000000000015763

**Published:** 2019-05-31

**Authors:** Chia-Chun Chen, Chin-Tse Lee, Shih-Lun Chang, Meng-Chen Tsai

**Affiliations:** a Department of Otorhinolaryngology, Chi Mei Medical Center; b Department of Optometry, Chung Hwa University of Medical Technology; c Pathology Center, Chi Mei Medical Center, Tainan, Taiwan.

**Keywords:** lung adenocarcinoma, tonsil neoplasm, tonsillar metastasis

## Abstract

**Rationale:**

Palatine tonsil is an extremely rare site for metastatic disease, accounting for 0.8% of malignant tonsillar neoplasms. To the best of our knowledge, this is the first report of metastatic adenocarcinoma in the tonsil treated with wide excision and targeted therapy, with no local recurrence 6 months postoperatively.

**Patient concerns:**

A 75-year-old man presented hemoptysis and mild productive cough for 2 weeks.

**Diagnoses:**

Palatine tonsil metastasis from lung adenocarcinoma, pT2bN0M1b, stage IVA, was confirmed.

**Interventions:**

Wide excision of primary lung tumor and metastatic tonsil carcinoma has been performed, and the patient was undergoing targeted therapy with the epidermal growth factor receptor inhibitor afatinib.

**Outcomes:**

There was no local recurrence in the oropharynx 6 months postoperatively.

**Lessons:**

We aim at highlighting the importance of a thorough evaluation for suspicion of tonsillar enlargement, which might be a sign of a primary malignancy elsewhere.

## 1 Introduction

Palatine tonsil is an extremely rare site for metastatic disease, accounting for 0.8% of malignant tonsillar neoplasms.^[1]^ To date, only 22 cases with primary lung origin have been reported, and only 1 case of lung adenocarcinoma with palatine tonsil metastasis was documented.^[2]^ Currently, there is no standard treatment and the prognosis is poor for tonsillar metastasis. Due to its rarity and significant effect on survival rate, we present a case of a 75-year-old male with palatine tonsil metastasis from lung adenocarcinoma.

## 2 Case report

A 75-year-old man was admitted with a history of hemoptysis and mild productive cough for 2 weeks. He had neither fever, weight loss, dyspnea, or dysphagia, nor pharyngeal foreign body sensation. His past medical and surgical history included hypertension and gastroesophageal reflux disease. He used to be a smoker and a betel nut chewer but had quit both for 15 years. There was neither history of ear, nose, and throat problems nor family history of such.

**Figure 1:**
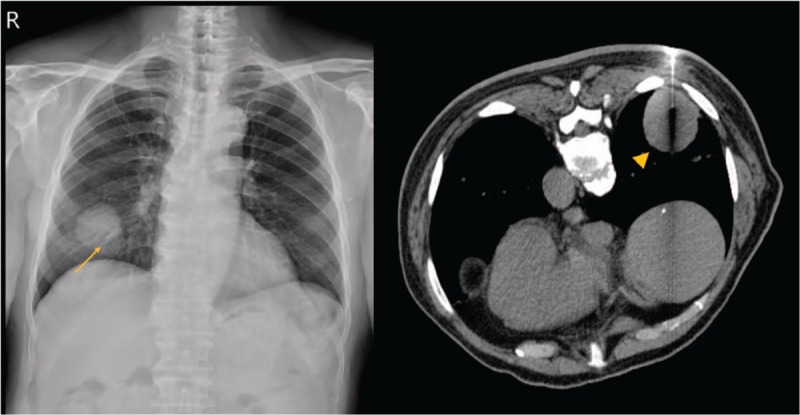
The chest X-ray showed opacity in right lower lung (arrow). Computed tomography (CT) scan revealed a mass of 5 × 4.1 cm in right lower lung (arrowhead).

Physical examination revealed mildly decreased breathing sounds on the right side. There were no remarkable findings in the head and neck region. Laboratory findings were within normal range. The chest X-ray showed opacity in right lower lung, and a subsequent computed tomography (CT) scan revealed a mass of 5 × 4.1 cm in right lower lung (Fig. 1) and small solid nodules in the right upper lung. The CT-guided lung biopsy of the right lower lobe showed necrotic atypical cells on histopathological examination. The patient underwent a video-assisted thoracoscopic surgery (VATS) right lower lobectomy with lymph nodes dissection and VATS wedge resection of right upper lobe, which confirmed moderately differentiated lung adenocarcinoma with epidermal growth factor receptor (EGFR) exon 19 deletions in the right lower lobe (pT2bN0) and minimally invasive, well-differentiated lung adenocarcinoma in the right upper lobe (pT1miN0).

**Figure 2:**
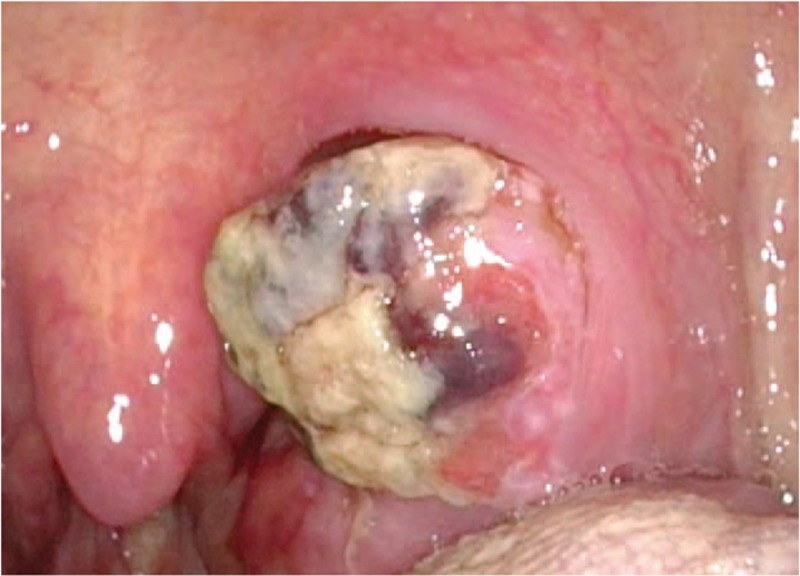
An exophytic with necrotic and hemorrhagic mass in the upper pole of left palatine tonsil.

**Figure 3:**
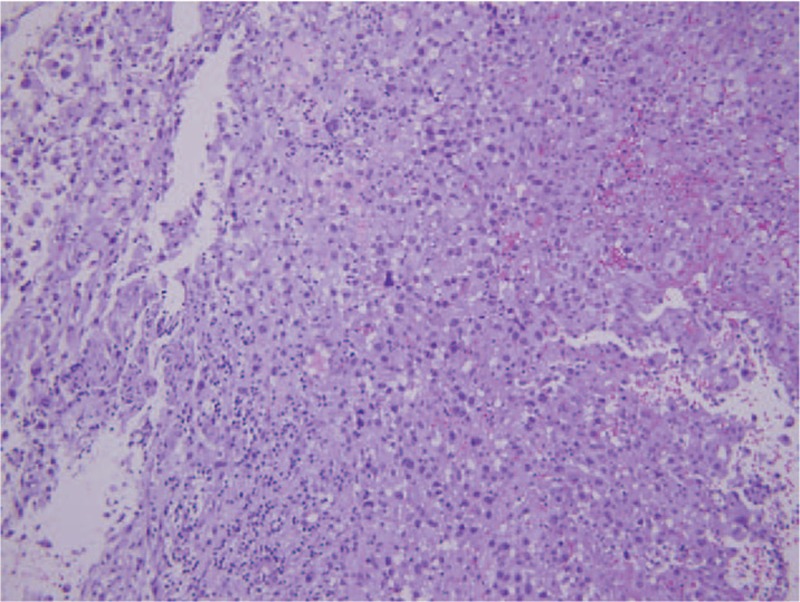
Microscopic finding of biopsy of left palatine tonsil tumor. Hematoxylin and eosin, 200×.

**Figure 4:**
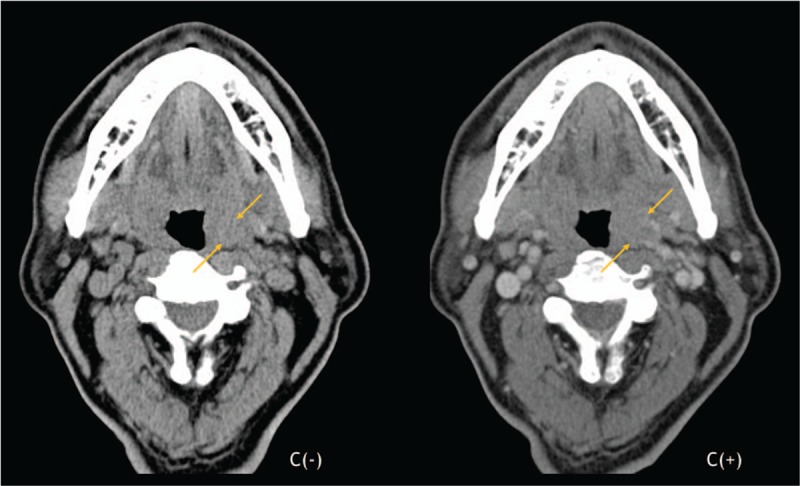
Neck computed tomography (CT) scan revealed a 29 × 20 mm homogenous lesion with enhancing soft tissue in the left palatine tonsil (arrow), without suspicious lymph node metastasis.

**Figure 5:**
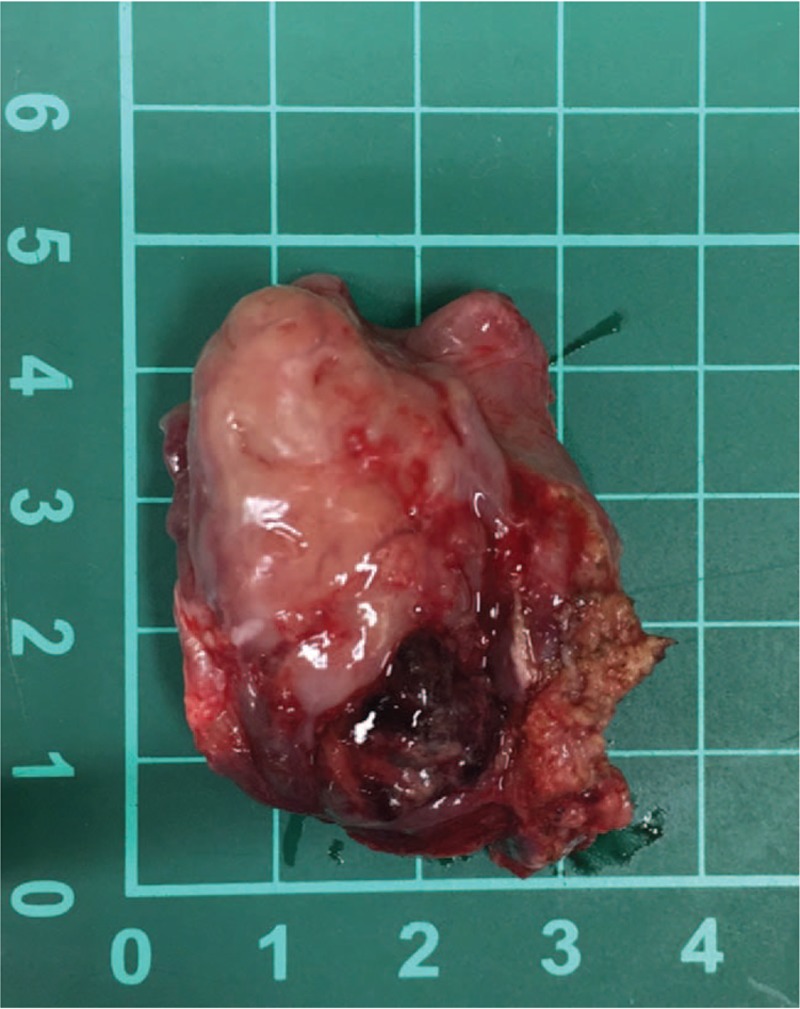
Gross view of wide excision of left palatine tonsil.

**Figure 6:**
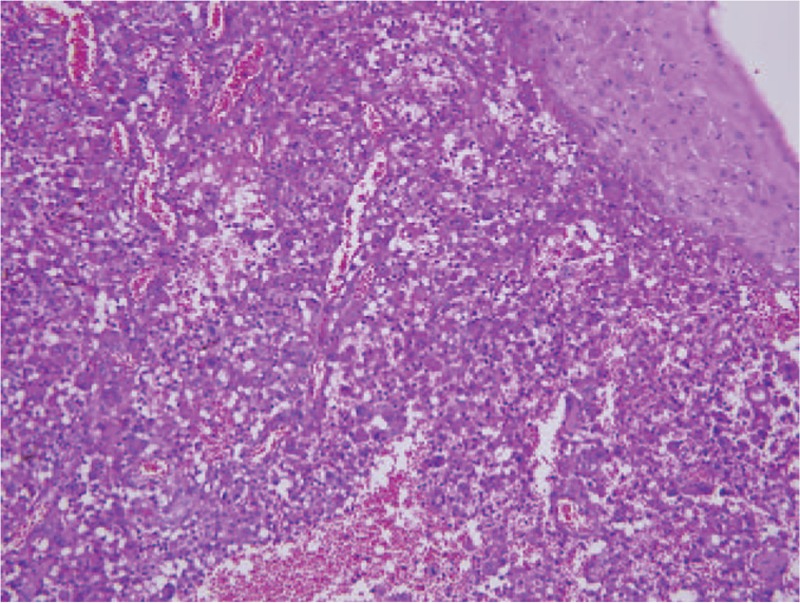
Microscopic finding of wide excision of left palatine tonsil. Hematoxylin and eosin, 200×.

Two months after first presentation, the patient still presented with hemoptysis. Head and neck examination revealed a mass in the upper pole of left palatine tonsil. The mass was exophytic with necrotic and hemorrhagic areas (Fig. 2). A biopsy confirmed carcinoma, positive for thyroid transforming factor-1 and AE1/AE3 but negative for p40 and thyroglobulin, which is consistent with the carcinoma of lung origin (Fig. 3). A neck CT scan revealed a 29 × 20 mm homogenous lesion with enhancing soft tissue in the left palatine tonsil, without suspicious lymph node metastasis (Fig. 4). Wide excision of the left palatine tonsil was performed (Fig. 5), and specimens of lung and tonsil shared morphological similarities in side-by-side comparison. The neoplastic cells are immunoreactive for thyroid transforming factor-1, cytokeratin-7, but not for p40 and cytokeratin-20, confirming metastatic adenocarcinoma with pulmonary origin (Fig. 6).

The final diagnosis was right lower lung adenocarcinoma with left oropharynx metastasis, pT2bN0M1b, stage IVA, with the follow-up period being 6 months till now. There was no local recurrence in the left oropharynx. The patient was undergoing targeted therapy with the EGFR inhibitor, afatinib 40 mg/d by oral administration for 3 months, and had regular follow up at the chest and otorhinolaryngology outpatient departments.

## 3 Discussion

Tonsillar metastasis is rare, accounting for 0.8% of malignant tonsillar neoplasms.^[1]^ The most common primary tumor sites are breast,^[2]^ stomach,^[3]^ colorectal tract,^[4,5]^ melanoma,^[6]^ and the kidney.^[3]^ Only 22 cases of lung cancer with tonsil metastasis have been reported in the literature, with 20 in the palatine tonsil and 2 in the lingual tonsil. Major histological types were small cell lung carcinoma.^[7]^ There was only 1 lung adenocarcinoma with palatine tonsil metastasis documented before by Mastronikolis et al.^[8]^

The metastatic pathway to the tonsil remains controversial. The hematogenous route may be responsible for most cases, especially for the intraabdominal primary tumors.^[7,8]^ As palatine tonsils have efferent lymphatic drainage, retrograde lymphatic spread to the tonsil has also been proposed but considered unusual.^[8,9]^ Spreading through the paravertebral plexus and by direct implantation of cancer cells from instrumentation during bronchoscopy have been suggested as well in patients with lung cancer.^[3,7]^

Image workup including neck CT is needed to evaluate the extent of the tonsillar mass and the status of the cervical lymph nodes, whereas diagnosis of tonsillar metastasis should be confirmed by pathological proof of tonsil biopsy. Surgical treatment could be conducted if the tonsillar mass is relative small, whereas radiotherapy or chemotherapy should be considered in addition to surgery in larger lesions.^[10]^

However, there is no standard and effective treatment for tonsillar metastasis from lung cancer. Thus, the prognosis is poor with mean survival being 9 months or less after the development of tonsillar metastasis, unrelated to the primary tumor.^[3]^ Recent reports indicate that EGFR inhibitors significantly improved prognosis of lung cancer with tonsillar metastasis, with a mean progression-free survival of 4.7 months by first and second-line regimens and 58.8 months with gefitinib and an overall survival of 82.4 months.^[11]^ The previous case of lung adenocarcinoma with tonsil metastasis reported by Mastronikolis et al was treated with radiotherapy, but the patient expired within weeks due to disseminated disease.^[8]^ In our case, the patient underwent surgery including VATS lobectomy of lung and wide excision of the tonsil, followed by the EGFR inhibitor, afatinib, due to exon 19 deletions. The patient had no local recurrence 6 months postoperatively.

This is the first report of metastatic adenocarcinoma in the tonsil treated with wide excision and targeted therapy, with no local recurrence 6 months postoperatively. As shown in the literature, when evaluating a suspicious tonsillar enlargement, clinicians need to be aware that the lesion could be the manifestation of primary malignancies elsewhere in the body, which is relatively uncommon and associated with lower survival rates.

## Author contributions

**Resources:** Chin-Tse Lee, Shih-Lun Chang, Meng-Chen Tsai.

**Supervision:** Shih-Lun Chang.

**Writing – original draft:** Chia-Chun Chen.

**Writing – review and editing:** Chia-Chun Chen, Shih-Lun Chang.

## References

[1] Hyams VJ Differential diagnosis of neoplasia of the palatine tonsil. Clin Otolaryngol Allied Sci 1978;3:117–26. 668170 10.1111/j.1365-2273.1978.tb00674.x

[2] Bar R Netzer A Ostrovsky D Abrupt tonsillar hemorrhage from a metastatic hemangiosarcoma of the breast: case report and literature review. Ear Nose Throat J 2011;90:116–20. 21412741 10.1177/014556131109000309

[3] Richmond Brownson William E Jaques Samuel E LaMonte Hypernephroma metastatic to the palatine tonsils. Ann Otol Rhinol Laryngol 1979;88 ((2 Pt 1)):235–40. 443718 10.1177/000348947908800215

[4] Sheng LM Zhang LZ Xu HM Ascending colon adenocarcinoma with tonsillar metastasis: a case report and review of the literature. World J Gastroenterol 2008;14:7138–40. 19084924 10.3748/wjg.14.7138 PMC2776847

[5] Guvenc MG Ada M Acioglu E Tonsillar metastasis of primary signet-ring cell carcinoma of the cecum. Auris Nasus Larynx 2006;33:85–8. 16169179 10.1016/j.anl.2005.07.009

[6] Aydogan LB Myers JN Myers EN Malignant melanoma metastatic to the tonsil. Laryngoscope 1996;106:313–6. 8614195 10.1097/00005537-199603000-00013

[7] Unsal M Kutlar G Sullu Y Tonsillar metastasis of small cell lung carcinoma. Clin Respir J 2016;10:681–3. 25620524 10.1111/crj.12275

[8] Mastronikolis NS Tsiropoulos GE Chorianopoulos D Palatine tonsillar metastasis from lung adenocarcinoma. Eur Rev Med Pharmacol Sci 2007;11:279–82. 18074935

[9] Arroyo HH Takehara J Ogawa AI Small cell lung carcinoma metastasis to palatine tonsils. Braz J Otorhinolaryngol 2013;79:645. 24141685 10.5935/1808-8694.20130117 PMC9442356

[10] Tsubochi H Isogami K Sato N Successfully treated lingual tonsillar from bronchial adenocarcinoma metastasis. Jpn J Thorac Cardiovasc Surg 2005;53:455–7. 16164261 10.1007/s11748-005-0085-8

[11] Gottschling S Penzel R Pelz T KRAS-mutation positive, metastatic tonsil carcinoma with cancer stem-like cell features and long-term response to gefitinib: a case report and review of the literature. J Clin Oncol 2011;29:e616–9. 21555681 10.1200/JCO.2011.34.5892

